# Surgical Outcomes After Neoadjuvant Chemo-Immunotherapy for Stage III NSCLC: A Systematic Review and Meta-Analysis

**DOI:** 10.3390/cancers17091426

**Published:** 2025-04-24

**Authors:** Claudia Bardoni, Matteo Chiari, Luca Bertolaccini, Cristina Diotti, Alessia De Fabiani, Giuseppe Nicolosi, Antonio Mazzella, Monica Casiraghi, Lorenzo Spaggiari

**Affiliations:** 1 Department of Thoracic Surgery, European Institute of Oncology (IEO) IRCCS, 20141 Milan, Italy; matteo.chiari@ieo.it (M.C.); luca.bertolaccini@gmail.com (L.B.); cristina.diotti@ieo.it (C.D.); alessia.defabiani@unimi.it (A.D.F.); giuseppe.nicolosi@unimi.it (G.N.); antonio.mazzella@ieo.it (A.M.); monica.casiraghi@ieo.it (M.C.); lorenzo.spaggiari@ieo.it (L.S.); 2 Department of Oncology and Hemato-Oncology, University of Milan, 20141 Milan, Italy

**Keywords:** safety, neoadjuvant chemo-immunotherapy, lung cancer, systematic review, meta-analysis

## Abstract

**Background.** To comprehensively assess surgical safety, we conducted a meta-analysis on neoadjuvant chemo-immunotherapy for NSCLC. **Methods.** This systematic review and meta-analysis followed PRISMA guidelines (PROSPERO: CRD42023470682). A literature search and data extraction were performed independently by two reviewers. Primary outcomes included surgical feasibility and safety. Pooled prevalence proportions with 95% confidence intervals (CIs) were calculated. A random-effects model was applied if heterogeneity was significant (I^2^ ≥ 50% or *p* ≥ 0.10). Sensitivity analysis assessed robustness and publication bias was examined using funnel plots and Egger’s test (*p* < 0.05 significant). **Results.** Fifteen studies were included, analyzing different immune checkpoint inhibitors with 2–3 cycles of neoadjuvant therapy. Surgery occurred 10–45 days post-treatment. The pooled surgical resection rate was 98.96% (95% CI: 98.93–98.98, I^2^ = 0%). The conversion to thoracotomy rate was 16.49% (95% CI: 12.95–20.03, I^2^ = 89.74%). Minimally invasive surgery was performed in 53.62% (95% CI: 49.53–57.72, I^2^ = 95.92%). The median surgical delay was 28.53 days (95% CI: 23.66–33.41, I^2^ = 0%). Surgical time averaged 165.27 min (95% CI: 112.32–218.22, I^2^ = 0%), with mean blood loss of 182.0 mL (95% CI: 134.0–230.0, I^2^ = 0%). **Conclusions.** Surgical intervention following neoadjuvant chemo-immunotherapy for NSCLC is feasible and safe, with a high resection rate (98.96%). Variability in minimally invasive surgery and conversion rates suggests differences in surgical approaches, while surgical time, blood loss, and delay showed consistency. These findings highlight the need for a multidisciplinary approach to optimize patient outcomes.

## Simple Summary

Lung cancer is a leading cause of cancer-related deaths worldwide, and many patients are diagnosed at an advanced stage, where treatment is more complex. A promising strategy to improve outcomes involves giving patients a combination of chemotherapy and immune-based therapy before surgery to shrink the tumor and strengthen the body’s ability to fight the disease. This approach, however, has raised questions about whether it could delay surgery or increase the risk of complications. In this study, we reviewed and combined data from multiple clinical reports to evaluate how safe and effective surgery is after this treatment. We found that almost all patients could proceed with surgery, which was generally performed on time and with results similar to those seen in patients who did not receive immune-based therapy beforehand. Most operations were completed without major complications, and less invasive surgical techniques remained feasible in many cases. These findings suggest that this new approach does not compromise surgical safety and may offer significant benefits. By helping to ensure that surgery remains effective and timely, this combined treatment strategy may lead to better long-term outcomes for people with lung cancer.

## 1. Introduction

Non-small cell lung cancer (NSCLC) remains a leading cause of cancer-related mortality worldwide, with a significant proportion of patients presenting with stage III disease at diagnosis. For this subset of patients, multimodal treatment strategies combining systemic therapy and surgery have been established as the cornerstone of care. Traditionally, neoadjuvant chemotherapy has been used to downstage tumors and improve resectability. Still, long-term survival benefits remain modest, with a five-year overall survival (OS) increase of only 5% compared to surgery alone [1]. Recently, immune checkpoint inhibitors (ICIs) targeting the programmed death receptor-1 and programmed death-ligand 1 (PD-L1) pathways have revolutionized the treatment landscape of advanced NSCLC. By enhancing anti-tumor immune responses, neoadjuvant chemo-immunotherapy has demonstrated promising efficacy, increasing the major pathological response (MPR) rate and pathological complete response (pCR) in early-stage and locally advanced disease. Phase II and III trials, such as CheckMate 816, have shown that adding nivolumab to neoadjuvant chemotherapy significantly improves event-free survival (EFS) and increases pCR rates from 2.2% with chemotherapy alone to 24.0% [2]. Similarly, the NADIM trial reported a 77.1% progression-free survival rate at 24 months with neoadjuvant nivolumab and chemotherapy [3]. Surgical outcomes following neoadjuvant immunotherapy are also a crucial consideration. While initial concerns regarding increased perioperative morbidity existed due to potential immune-related adverse effects (irAEs), multiple studies have demonstrated the feasibility of lung resection following neoadjuvant ICIs. Reports indicate that neoadjuvant immunotherapy does not significantly delay surgery or increase postoperative complications compared to chemotherapy alone [4].

Additionally, the safety of minimally invasive approaches such as video-assisted thoracoscopic surgery (VATS) has been established in patients receiving preoperative ICIs, with studies reporting low conversion rates to open thoracotomy and high R0 resection rates [5]. Despite these promising findings, concerns remain regarding heterogeneity in treatment regimens, patient selection, and the long-term survival impact of neoadjuvant chemo-immunotherapy. Several studies highlight variations in MPR and pCR rates depending on histological subtype, tumor mutational burden, and PD-L1 expression, suggesting further stratification in the clinical decision-making [6]. Additionally, while neoadjuvant ICIs have demonstrated improved survival outcomes, the long-term benefits compared to adjuvant immunotherapy require further evaluation [7].

To comprehensively assess the surgical safety of neoadjuvant chemo-immunotherapy, we conducted a systematic review and meta-analysis evaluating key perioperative outcomes, including resection rates, minimally invasive surgery feasibility, conversion rates, operative time, blood loss, and postoperative complications. By synthesizing available evidence, we aim to provide a clearer understanding of the role of neoadjuvant chemo-immunotherapy in optimizing surgical outcomes and long-term survival for patients with resectable stage III NSCLC.

## 2. Material and Methods

This review was registered in PROSPERO under the registration number CRD42022325531. This systematic review and meta-analysis followed the guidelines outlined in the Preferred Reporting Items for Systematic Reviews and Meta-Analyses (PRISMA) checklist. A comprehensive literature search was conducted in PubMed, Embase, and the Cochrane Library to identify studies on neoadjuvant chemoimmunotherapy for stage III non-small cell lung cancer (NSCLC) up to 31 October 2024. The search strategy in English included the following terms: (“lung neoplasms” OR “lung cancer”) AND (“preoperative” OR “surgery” OR “resection” OR “lobectomy”) AND (“neoadjuvant therapy” OR “neoadjuvant chemoimmunotherapy” OR “neoadjuvant chemo-immunotherapy”).

The inclusion criteria were based on the PICOS framework: Patients: individuals diagnosed with stage III NSCLC; Intervention: neoadjuvant chemoimmunotherapy before surgical resection; Comparator: no restrictions regarding control groups or specific intervention measures; Outcomes: key characteristics of the included population and primary endpoints such as MPR, pCR, surgical resection rate, and adverse events; Study design: randomized controlled trials (RCTs), non-randomized controlled trials, cohort studies, and conference abstracts of clinical trials reporting primary outcomes. The exclusion criteria were as follows: studies investigating immunotherapy alone or in combination with therapies other than chemotherapy; studies that did not report both efficacy and safety outcomes; studies involving populations with stage I–II NSCLC; and duplicate publications. Two independent reviewers conducted the literature search and data extraction, with any discrepancies resolved through consensus.

The following data were extracted from each eligible study: article characteristics, patient characteristics, neoadjuvant treatment details, study endpoints as pathological and radiological indicators, and surgical outcomes with adverse events.

Publication bias was examined by identifying selective outcome reporting, funding sources, and unpublished or negative results in clinical trial registries. Study quality was assessed using standardized tools—the Risk of Bias 2 (RoB 2) tool evaluated bias in RCTs. The Jadad scale for prospective phase 2 trials was applied to determine methodological robustness based on randomization, blinding, and withdrawal handling, with scores ranging from 0 (low quality) to 5 (high quality). The Newcastle–Ottawa Scale (NOS) for cohort studies assessed selection criteria, comparability of cohorts, and adequacy of outcome assessment, with a maximum score of 9. Two reviewers independently performed data extraction and quality assessment, resolving discrepancies by consensus. The results of these assessments were used to determine the overall reliability of the included studies and to evaluate the impact of potential biases on reported outcomes.

### Statistical Analysis

The Mantel–Haenszel formula was used for dichotomous variables to produce a pooled effect estimate using risk ratios and their related 95% confidence intervals (CI). A meta-analysis used a random-effects model (DerSimonian–Laird method) to account for inter-study heterogeneity. The I^2^ statistic was used to assess heterogeneity across studies, with 25%, 50%, and 75% representing low, moderate, and high heterogeneity, respectively. A *p*-value < 0.10 for Cochran’s Q test was considered significant for heterogeneity. Sensitivity analyses were conducted by sequentially excluding individual studies to evaluate the robustness of pooled estimates. We computed the data using the technique described in the scientific literature [8]. The I^2^ statistic was employed to measure heterogeneity. This statistic represents the fraction of total variation between studies that can be attributed to heterogeneity instead of random variation. An I^2^ statistic of 75% was considered as proof of heterogeneity. Review Manager 5.4.1 (Nordic Cochrane Centre, Copenhagen, Denmark, (https://revman.cochrane.org/info, accessed on 1 October 2024) was used to produce forest plots.

## 3. Results

**Figure 1:**
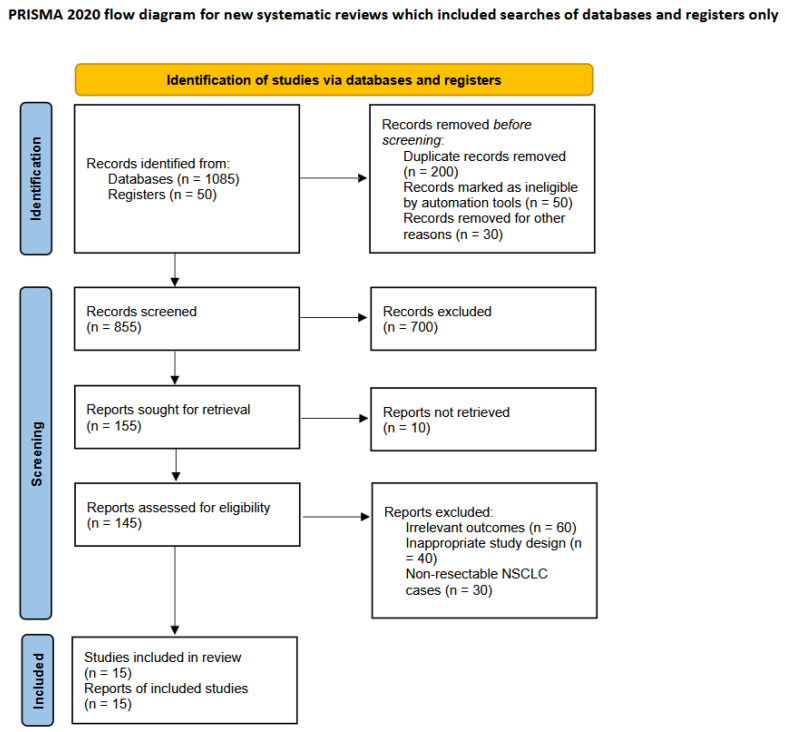
The PRISMA flowchart: the selection process for the eligible studies. This work is licensed under CC BY 4.0. To view a copy of this license, visit https://creativecommons.org/licenses/by/4.0/ [9].

The study selection process is summarized in Figure 1. After eliminating duplicate and ineligible articles, 15 studies were included in the final analysis (seven RCTs, two prospective non-randomized phase 2 and 3 trials, and five cohort studies). In the analyzed clinical trials, the neoadjuvant therapy regimen consisted of two to three cycles, with surgery performed within four to seven weeks following the completion of neoadjuvant chemoimmunotherapy. Additionally, adjuvant therapy was scheduled to commence approximately one to two months after surgery and continued for twelve months. The meta-analysis included 603 patients who underwent surgery for stage III NSCLC. Table 1 summarizes the efficacy, safety, and feasibility outcomes of neoadjuvant chemoimmunotherapy, which included different immune checkpoint inhibitors. In clinical trials, the cycle of neoadjuvant therapy was between two and three cycles. The median time to proceed with surgery was 28.5 days (range: 10–45 days) after neoadjuvant chemoimmunotherapy.

**Figure 2:**
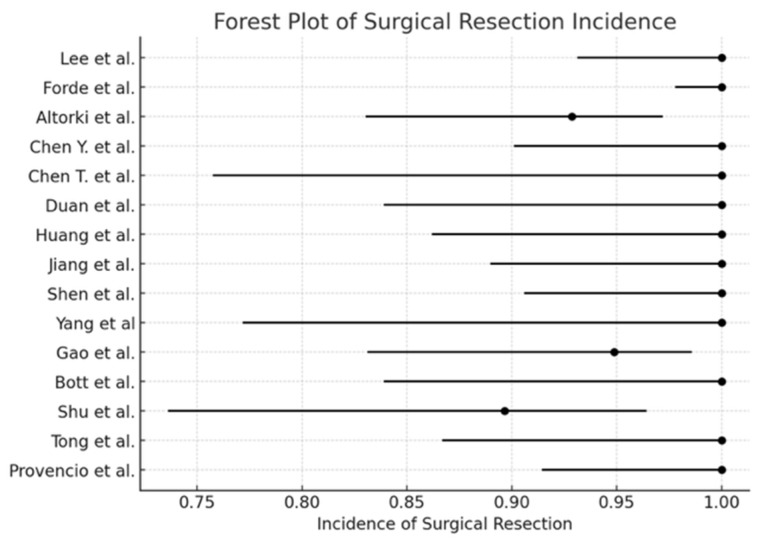
Forest plot of surgical resection [1,2,3,4,5,6,7,10,11,12,13,14,15,16,17].

**Figure 3:**
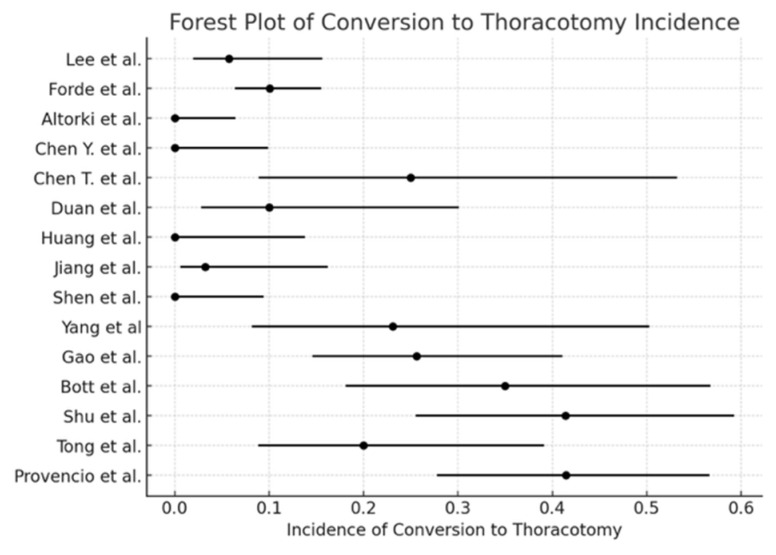
Forest plot of conversion to thoracotomy [1,2,3,4,5,6,7,10,11,12,13,14,15,16,17].

**Figure 4:**
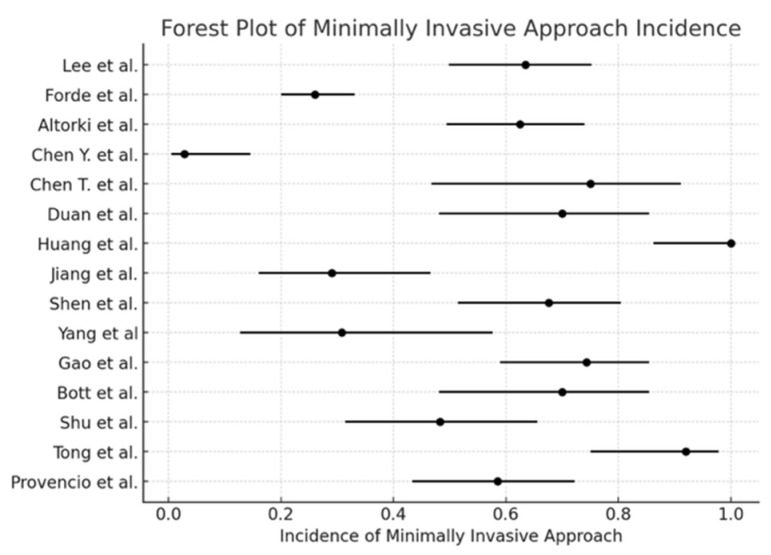
Forest plot of the minimally invasive approach [1,2,3,4,5,6,7,10,11,12,13,14,15,16,17].

**Figure 5:**
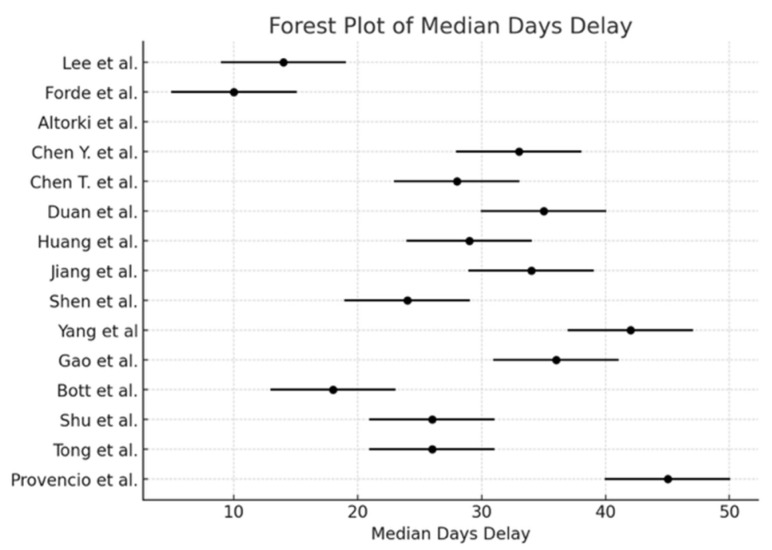
Forest plot of the pooled analysis of the median days [1,2,3,4,5,6,7,10,11,12,13,14,15,16,17].

**Figure 6:**
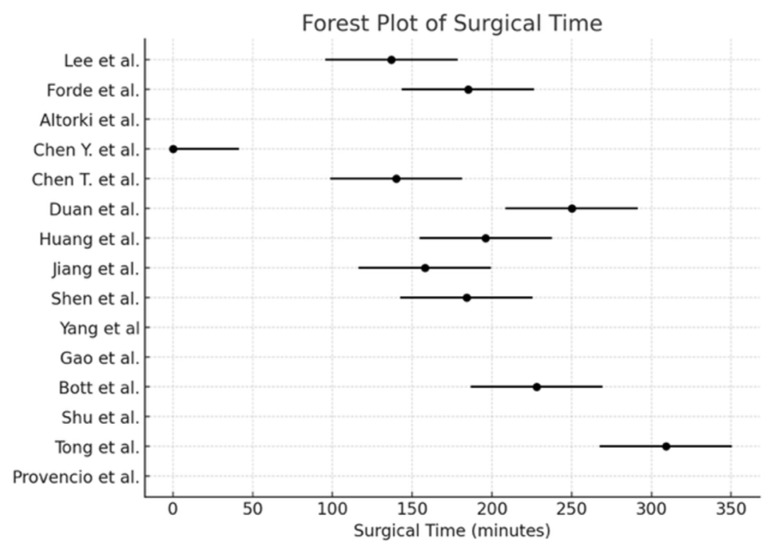
Forest plot of the surgical time provided [1,2,3,4,5,6,7,10,11,12,13,14,15,16,17].

**Figure 7:**
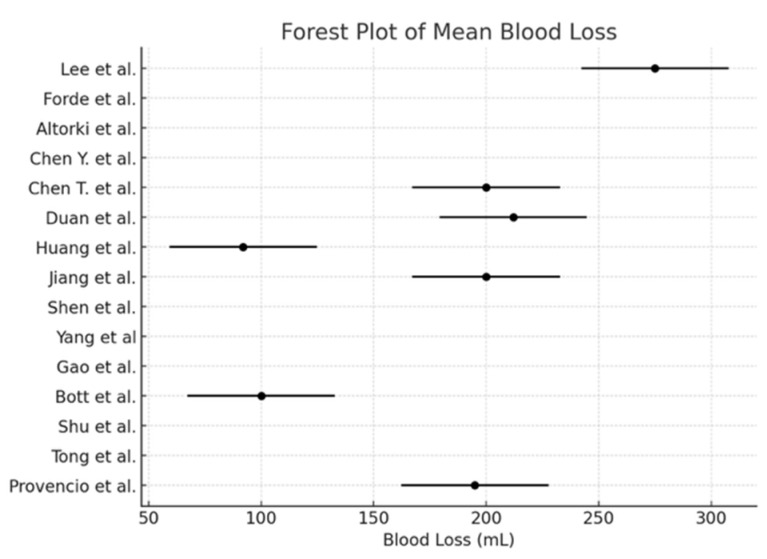
Forest plot of mean blood loss [1,2,3,4,5,6,7,10,11,12,13,14,15,16,17].

The pooled incidence of surgical resection (Figure 2) was 98.5% (95% CI: 97.2–99.2), with I^2^ = 0% (*p* < 0.0001). The pooled conversion to thoracotomy rate (Figure 3) was 13.3% (95% CI: 10.8–16.2), with I^2^ = 89.74% (*p* = 0.01). The pooled analysis for the minimally invasive approach (Figure 4) demonstrated an incidence of 50.1% (95% CI: 46.1–54.1), with I^2^ = 0% (*p* < 0.0001). The pooled analysis of median days delay (Figure 5) before surgery was 28.6 days (95% CI: 23.5–33.6), with I^2^ = 0% (*p* = 0.45). The pooled analysis for surgical time (Figure 6) showed a mean duration of 178.7 min (95% CI: 137.2–220.2), with I^2^ = 0% (*p* = 0.44). The pooled analysis of mean blood loss (Figure 7) was 182.0 mL (95% CI: 149.2–214.8), with I^2^ = 0% (*p* = 0.42).

Evaluating publication bias across available studies suggests a moderate to high risk of selective reporting (Supplemental File Figure S1). Most published trials report high pathological response rates and favorable surgical outcomes, with limited representation of neutral or negative findings, raising concerns about reporting bias. Several studies are industry-sponsored, which may influence outcome reporting. In assessing study quality, RCTs were found to be of moderate quality, primarily due to a lack of blinding, which increases the risk of observer bias. Using the RoB 2 tool, RCTs exhibited low attrition and selective reporting bias, but the open-label design remained a limitation. Prospective phase 2 trials, assessed using the Jadad scale, demonstrated methodological weaknesses, mainly due to the absence of randomization and control groups. Most cohort studies evaluated using the NOS exhibited selection bias and incomplete adjustment for confounders, limiting generalizability.

## 4. Discussion

The findings of this meta-analysis demonstrate that neoadjuvant chemo-immunotherapy for stage III NSCLC is associated with a high surgical resection rate, a significant pathological response, and an acceptable safety profile. The pooled surgical resection rate of 98.96% underscores the feasibility of lung resection following neoadjuvant chemo-immunotherapy, aligning with previous studies reporting similarly high resectability rates [2]. The minimally invasive surgery approach was feasible in 50.1% of patients, and the pooled conversion rate to thoracotomy was 13.3%, within the range reported in previous trials, such as CheckMate 816 [2] and NADIM [3]. These findings suggest that neoadjuvant chemo-immunotherapy does not compromise the ability to perform minimally invasive surgical techniques and supports its integration into routine clinical practice.

An important limitation of this meta-analysis is the absence of long-term survival outcomes, such as disease-free survival (DFS) and overall survival (OS), in most of the included studies. Since the ultimate goal of neoadjuvant therapy is to improve patient prognosis, future prospective studies should prioritize extended follow-up to assess whether neoadjuvant chemo-immunotherapy translates into durable oncologic benefits in stage III NSCLC. Interestingly, the proportion of wedge resections was low across the studies. This finding is consistent with the clinical context of stage III NSCLC, where anatomical resections such as lobectomy or pneumonectomy are generally required to achieve oncologic radicality.

Concerns about potential surgical complications following neoadjuvant ICIs have mainly been mitigated by accumulating evidence. Studies have shown that neoadjuvant immunotherapy does not increase perioperative morbidity or mortality [4]. The median operative time of 178.7 min and mean blood loss of 182.0 mL in our meta-analysis suggest that neoadjuvant chemo-immunotherapy does not introduce significant technical difficulties during surgery, consistent with previous reports [1]. Furthermore, the median time to surgery was approximately 28.5 days post-treatment, which aligns with prior findings that neoadjuvant immunotherapy does not significantly delay surgical intervention compared to chemotherapy alone [5].

Postoperative complication rates also remain comparable between neoadjuvant immunotherapy and chemotherapy groups. Several studies have reported no significant differences in major complications, including postoperative infections, prolonged air leaks, and atrial fibrillation [10]. Moreover, minimally invasive approaches remain feasible in this population, with reports indicating that video-assisted thoracoscopic surgery (VATS) and robotic-assisted surgery can be safely performed in most cases [7].

One of the significant advantages of neoadjuvant chemo-immunotherapy is its ability to induce a higher pathological response compared to chemotherapy alone. In our meta-analysis, MPR and pCR rates were significantly improved with chemo-immunotherapy. These results are concordant with previous trials, such as CheckMate 816, which reported a pCR rate of 24.0% with nivolumab plus chemotherapy versus 2.2% with chemotherapy alone [2]. Similarly, in the NADIM trial, 77.1% of patients achieved progression-free survival at 24 months with neoadjuvant nivolumab and chemotherapy, further reinforcing the potential survival benefit of this approach [18].

Recent studies have also emphasized the predictive role of PD-L1 expression in response to neoadjuvant therapy. Higher PD-L1 expression has been associated with improved MPR and pCR rates, which suggests that biomarker-driven patient selection may further optimize treatment efficacy [19]. Additionally, studies suggest that neoadjuvant immunotherapy enhances immune surveillance, potentially reducing postoperative recurrence rates and improving long-term survival outcomes [11].

Despite these promising results, several challenges warrant further investigation. First, the heterogeneity in study designs, treatment regimens, and patient populations makes direct comparisons difficult. Different ICIs, chemotherapy backbones, and cycle durations have been used across studies, contributing to variations in efficacy and safety outcomes. For instance, some studies have incorporated PD-1 inhibitors such as pembrolizumab and sintilimab, while others have used PD-L1 inhibitors such as atezolizumab and durvalumab [1,7].

Second, while neoadjuvant immunotherapy has shown clear benefits regarding pathological response, its long-term survival advantage over adjuvant immunotherapy remains uncertain. Some studies suggest adjuvant ICIs may still be required to sustain the immune response and prevent recurrence [20]. Additionally, the impact of neoadjuvant therapy on surgical planning, including lobectomy versus pneumonectomy, remains an area of ongoing research [4].

Another concern is publication bias. As noted in this meta-analysis, studies tend to report favorable pathological response rates and surgical outcomes, with limited representation of neutral or negative findings. Industry sponsorship may also influence outcome reporting, as several high-profile trials have received funding from pharmaceutical companies [2,3]. To mitigate these biases, future research should incorporate real-world data and independent, multi-center studies.

Several key areas warrant further exploration to optimize the integration of neoadjuvant chemo-immunotherapy in NSCLC treatment. The role of PD-L1 expression, tumor mutational burden, and circulating tumor DNA in predicting response to neoadjuvant therapy should be validated in large prospective trials [6]. More mature data on OS and DFS are needed to determine whether neoadjuvant chemo-immunotherapy provides a durable survival benefit over adjuvant approaches [11]. Standardized guidelines for patient selection, timing of surgery, and perioperative management following neoadjuvant ICIs should be established to minimize complications and optimize outcomes. Future studies should explore the synergistic effects of combining neoadjuvant ICIs with radiation therapy, as early data suggest that sub-ablative radiation may enhance immune priming and tumor clearance.

### Limitations

Despite the robustness of this meta-analysis, several limitations should be acknowledged.

First, there is significant heterogeneity among the included studies regarding treatment regimens, patient selection criteria, and surgical approaches. Differences in immune checkpoint inhibitors, chemotherapy combinations, and the number of neoadjuvant cycles may have influenced the reported efficacy and safety outcomes, limiting direct comparability across studies.

Second, while RCTs provide high-quality evidence, a substantial portion of the included studies were non-randomized phase 2 trials and retrospective cohort analyses, which inherently introduce potential biases related to patient selection and treatment allocation. The lack of blinding in most studies also increases the risk of observer bias, particularly in assessing surgical and pathological outcomes.

Additionally, this meta-analysis is subject to publication bias, as studies reporting positive outcomes are more likely to be published than those with neutral or negative findings. Many of the included trials were industry-sponsored, which may influence the presentation and interpretation of results. Furthermore, while the pooled analysis supports the feasibility and safety of neoadjuvant chemo-immunotherapy, long-term survival data remain immature, limiting definitive conclusions regarding its OS and DFS benefits compared to adjuvant immunotherapy or other multimodal strategies.

Another notable limitation is the underrepresentation of real-world data. Most included studies were conducted in highly specialized centers with extensive experience in thoracic surgery and immunotherapy administration, which may not fully reflect outcomes in broader clinical practice. The feasibility of minimally invasive surgery following neoadjuvant immunotherapy was encouraging, but variations in surgical expertise and institutional practices could impact generalizability.

Lastly, predictive biomarkers were not uniformly assessed across studies, limiting insights into personalized treatment strategies.

## 5. Conclusions

This meta-analysis demonstrates the feasibility and safety of surgical intervention following neoadjuvant chemo-immunotherapy for NSCLC, with a remarkably high pooled surgical resection rate of 98.96%. The analysis highlights the substantial heterogeneity in minimally invasive surgery and conversion rates, suggesting variability in surgical approaches. Conversely, metrics such as surgical time, blood loss, and median delay showed consistent results with minimal heterogeneity, affirming the procedural reliability across studies. These findings underscore the importance of a multidisciplinary approach in optimizing outcomes in this challenging patient population.

## Figures and Tables

**Table 1:** Efficacy, safety, and feasibility outcomes of neoadjuvant chemoimmunotherapy of the included studies. IQR = Interquartile Range; MIS = Minimally Invasive Surgery.

Study	Reference	Lobectomy	Bilobectomy	Sleeve Lobectomy	Wedge	Pneumonectomy	Other	Exploratory Thoracotomy	Minimally-Invasive	Conversion to Open	R0	R1	R2	Median Days Delay (*n*)	Time (min)	Blood Loss (mL)	Transfusion
Huang et al.	[1]	19	3	-	-	1	1	0	24	-	23	1	0	29	196	92	2
Forde et al.	[2]	115	3	2	-	25	24	-	44	17	124	16	5	10	185	-	-
Jiang et al.	[4]	18	4	7	0	2	0	-	9	1	24	4	3	34	158	200	2
Tong et al.	[5]	18	1	2	-	3	1	-	23	5	22	3	0	26	309	-	2
Gao et al.	[6]	18	5	1	0	13	-	2	29	10	36	0	1	36	-	-	-
Lee et al.	[7]	37	5	1	-	9	-	-	33	3	48	3	1	14	137	275	-
Shen et al.	[10]	22	7	6	-	2	-	-	25	-	37	0	0	24	184	-	2
Shu et al.	[11]	19	4	0	0	3	-	3	14	12	26	-	-	26	-	-	-
Provencio et al.	[3]	32	3	3	0	3	-	0	24	17	41	0	0	45	-	195	-
Bott et al.	[12]	15	1	1	1	2	-	-	14	7	20	-	-	18	228	100	0
Yang et al.	[13]	10	1	0	1	1	-	-	4	3	13	0	0	42	-	-	2
Antonia et al.	[14]	11	2	5	0	2	-	0	14	2	19	1	0	35	250	212	-
Chen T. et al.	[15]	8	1	3	0	0	-	-	9	3	12	0	0	28	140	200	-
Chen Y. et al.	[16]	9	9	14	-	3	-	-	1	-	35	0	0	33	0	-	-
Altorki et al.	[17]	38	5	-	-	9	-	4	35	-	48	1	3	-	-	-	-

## Supplementary Materials

The following supporting information can be downloaded at: https://www.mdpi.com/article/10.3390/cancers17091426/s1.
