# Supplementary figures and images for: Surgical Outcomes After Neoadjuvant Chemo-Immunotherapy for Stage III NSCLC: A Systematic Review and Meta-Analysis
Source: Cancers (Basel). 2025 Apr 24;17(9):1426. doi: 10.3390/cancers17091426 (PMC12070967; doi:10.3390/cancers17091426)

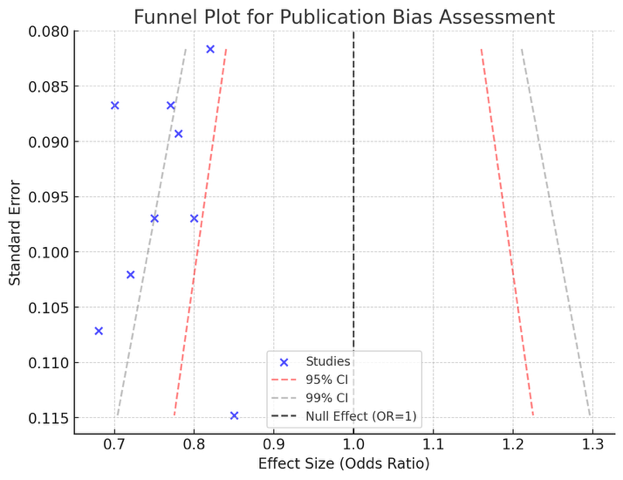

Supplement: Supplementary file 1 [file cancers-17-01426-s001.zip › cancers-3561053-Supplementary Figure.png]
